# *N*′-(2-Hydr­oxy-5-chloro­benzyl­idene)-4-nitro­benzohydrazide methanol solvate

**DOI:** 10.1107/S160053680800843X

**Published:** 2008-04-02

**Authors:** Ling Han, Shan-Shan Huang, Qing-Bai Huang, Xue-Mei Zhou, Yun-Peng Diao

**Affiliations:** a Liao Ning Benxi Third Pharmaceuticals Co Ltd, Benxi 117004, People’s Republic of China; b NERC for the Pharmaceuties of Traditional Chinese Medicines, Benxi 117004, People’s Republic of China; c College of Pharmacy, Dalian Medical University, Dalian 116044, People’s Republic of China

## Abstract

The title compound, C_14_H_10_ClN_3_O_4_·CH_4_O, was synthesized from the reaction of 5-chloro­salicylaldehyde with 4-nitro­benzohydrazide in methanol. The Schiff base mol­ecule is nearly planar, with a dihedral angle of 9.1 (3)° between the two benzene rings. The methanol solvent mol­ecules are linked to the Schiff base mol­ecules by N—H⋯O, O—H⋯N and O—H⋯O hydrogen bonds, forming chains running parallel to the *a* axis.

## Related literature

For related structures, see: Brückner *et al.* (2000 ▶); Diao (2007 ▶); Diao, Huang *et al.* (2008 ▶); Diao, Shu *et al.* (2007 ▶); Diao, Zhen *et al.* (2008 ▶); Harrop *et al.* (2003 ▶); Huang *et al.* (2007 ▶); Li *et al.* (2007 ▶); Ma *et al.* (2008 ▶); Ren *et al.* (2002 ▶); Wang *et al.* (2008 ▶).
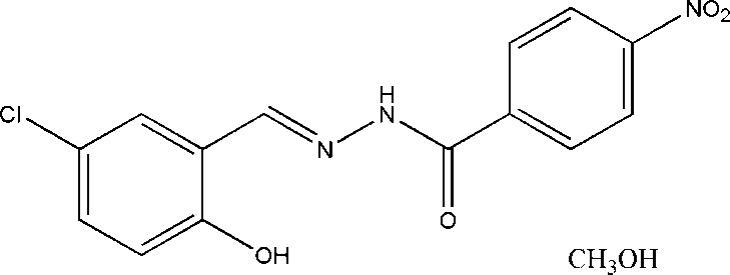


## Experimental

### 

#### Crystal data

C_14_H_10_ClN_3_O_4_·CH_4_O
*M* *_r_* = 351.74
Monoclinic, 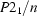

*a* = 6.628 (1) Å
*b* = 18.980 (3) Å
*c* = 12.521 (2) Å
β = 91.29 (3)°
*V* = 1574.7 (4) Å^3^
*Z* = 4
Mo *K*α radiation
μ = 0.27 mm^−1^
*T* = 298 (2) K
0.20 × 0.18 × 0.17 mm

#### Data collection

Bruker SMART CCD area-detector diffractometer
Absorption correction: multi-scan (*SADABS*; Bruker, 2000 ▶) *T*_min_ = 0.947, *T*_max_ = 0.955
9258 measured reflections
3259 independent reflections
1776 reflections with *I* > 2σ(*I*)
*R*_int_ = 0.056

#### Refinement

*R*[*F* ^2^ > 2σ(*F* ^2^)] = 0.055
*wR*(*F* ^2^) = 0.147
*S* = 1.01
3259 reflections
223 parameters
1 restraint
H atoms treated by a mixture of independent and constrained refinement
Δρ_max_ = 0.23 e Å^−3^
Δρ_min_ = −0.29 e Å^−3^

### 

Data collection: *SMART* (Bruker, 2000 ▶); cell refinement: *SAINT* (Bruker, 2000 ▶); data reduction: *SAINT*; program(s) used to solve structure: *SHELXTL* (Sheldrick, 2008 ▶); program(s) used to refine structure: *SHELXTL*; molecular graphics: *SHELXTL*; software used to prepare material for publication: *SHELXTL*.

## Supplementary Material

Crystal structure: contains datablocks global, I. DOI: 10.1107/S160053680800843X/ci2574sup1.cif

Structure factors: contains datablocks I. DOI: 10.1107/S160053680800843X/ci2574Isup2.hkl

Additional supplementary materials:  crystallographic information; 3D view; checkCIF report

## Figures and Tables

**Table 1:** Hydrogen-bond geometry (Å, °)

*D*—H⋯*A*	*D*—H	H⋯*A*	*D*⋯*A*	*D*—H⋯*A*
O4—H4⋯N3	0.82	2.04	2.745 (3)	144
O4—H4⋯O5^i^	0.82	2.47	2.930 (3)	116
O5—H5⋯O3^ii^	0.82	1.88	2.692 (3)	171
N2—H2*A*⋯O5	0.899 (10)	2.016 (13)	2.888 (3)	163 (3)

Symmetry codes: (i) 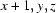; (ii) 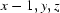.

**Fig. 1.**
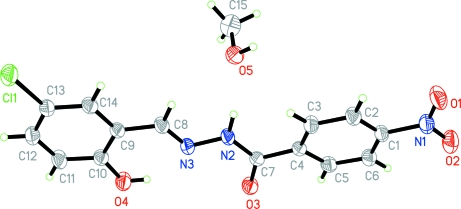
The molecular structure of the title compound with displacement ellipsoids drawn at the 30% probability level.

**Fig. 2.**
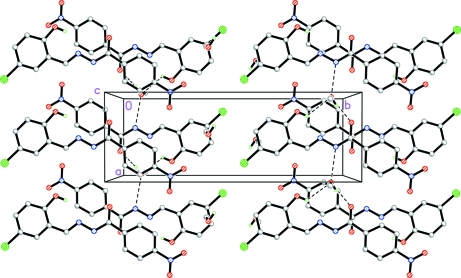
Crystal packing of the compound viewed along the c axis. Intermolecular hydrogen bonds are shown as dashed lines. H atoms not involved in the interactions have been omitted.

## supplementary crystallographic information

### Comment

Schiff base compounds have been found to have potential pharmacological and antitumor properties (Brückner *et al.*, 2000; Harrop *et al.*, 2003; Ren *et al.*, 2002). Recently, a few Schiff base compounds derived from the reaction of aldehydes with benzohydrazides have been reported (Diao 2007; Diao, Huang *et al.*, 2008; Diao, Shu *et al.*, 2007; Diao, Zhen *et al.*, 2008; Huang *et al.*, 2007; Li *et al.*, 2007; Ma *et al.*, 2008; Wang *et al.*, 2008). As a further study of such compounds, we report here the crystal structure of the title compound.

The asymmetric unit of the title compound consists of a Schiff base molecule and a lattice methanol molecule. The Schiff base molecule is nearly planar with the dihedral angle between the two benzene rings of 9.1 (3)°. The dihedral angle between the C1-C6 benzene ring and the O1/N1/O2 nitryl plane is 6.4 (3) °. The torsion angles C9—C8—N3—N2 and C4—C7—N2—N3 are 179.8 (2)° and -173.6 (2)°, respectively. The methanol solvent molecules are linked to the Schiff base molecules by N—H···O, O—H···N and O—H···O hydrogen bonds (Table 1), forming chains running along the *a* axis (Fig. 2).

### Experimental

5-Chlorosalicylaldehyde (0.1 mmol, 15.7 mg) and 4-nitrobenzohydrazide (0.1 mmol, 18.1 mg) were dissolved in a methanol solution (20 ml). The mixture was stirred at reflux for 1 h and cooled to room temperature. After keeping the solution in air for a week, yellow block-like crystals were formed.

### Refinement

Atom H2A was located from a difference Fourier map and refined isotropically, with the N–H distance restrained to 0.90 (1) Å. All other H atoms were placed in idealized positions and constrained to ride on their parent atoms, with C–H = 0.93–0.96 Å, O–H = 0.82 Å, and with *U*_iso_(H) = 1.2*U*_eq_(C) or 1.5*U*_eq_(O and methyl C).

### Crystal data

**Table:**

C_14_H_10_ClN_3_O_4_·CH_4_O	*F*_000_ = 728
*M**_r_* = 351.74	*D*_x_ = 1.484 Mg m^−^^3^
Monoclinic, *P*2_1_/*n*	Mo *K*α radiation λ = 0.71073 Å
Hall symbol: -P 2yn	Cell parameters from 1014 reflections
*a* = 6.628 (1) Å	θ = 2.5–24.3º
*b* = 18.980 (3) Å	µ = 0.27 mm^−^^1^
*c* = 12.521 (2) Å	*T* = 298 (2) K
β = 91.29 (3)º	Block, yellow
*V* = 1574.7 (4) Å^3^	0.20 × 0.18 × 0.17 mm
*Z* = 4	

### Data collection

**Table:**

Bruker SMART CCD area-detector diffractometer	3259 independent reflections
Radiation source: fine-focus sealed tube	1776 reflections with *I* > 2σ(*I*)
Monochromator: graphite	*R*_int_ = 0.056
*T* = 298(2) K	θ_max_ = 26.5º
ω scans	θ_min_ = 2.0º
Absorption correction: multi-scan(SADABS; Bruker, 2000)	*h* = −8→7
*T*_min_ = 0.947, *T*_max_ = 0.955	*k* = −22→23
9258 measured reflections	*l* = −15→15

### Refinement

**Table:**

Refinement on *F*^2^	Secondary atom site location: difference Fourier map
Least-squares matrix: full	Hydrogen site location: inferred from neighbouring sites
*R*[*F*^2^ > 2σ(*F*^2^)] = 0.055	H atoms treated by a mixture of independent and constrained refinement
*wR*(*F*^2^) = 0.147	*w* = 1/[σ^2^(*F*_o_^2^) + (0.0624*P*)^2^] where *P* = (*F*_o_^2^ + 2*F*_c_^2^)/3
*S* = 1.01	(Δ/σ)_max_ = 0.001
3259 reflections	Δρ_max_ = 0.23 e Å^−^^3^
223 parameters	Δρ_min_ = −0.29 e Å^−^^3^
1 restraint	Extinction correction: none
Primary atom site location: structure-invariant direct methods	

### Special details

**Table:**

Geometry. All e.s.d.'s (except the e.s.d. in the dihedral angle between two l.s. planes) are estimated using the full covariance matrix. The cell e.s.d.'s are taken into account individually in the estimation of e.s.d.'s in distances, angles and torsion angles; correlations between e.s.d.'s in cell parameters are only used when they are defined by crystal symmetry. An approximate (isotropic) treatment of cell e.s.d.'s is used for estimating e.s.d.'s involving l.s. planes.
Refinement. Refinement of *F*^2^ against ALL reflections. The weighted *R*-factor *wR* and goodness of fit *S* are based on *F*^2^, conventional *R*-factors *R* are based on *F*, with *F* set to zero for negative *F*^2^. The threshold expression of *F*^2^ > σ(*F*^2^) is used only for calculating *R*-factors(gt) *etc*. and is not relevant to the choice of reflections for refinement. *R*-factors based on *F*^2^ are statistically about twice as large as those based on *F*, and *R*- factors based on ALL data will be even larger.

### Fractional atomic coordinates and isotropic or equivalent isotropic displacement parameters (Å^2^)

**Table:**

	*x*	*y*	*z*	*U*_iso_*/*U*_eq_	
Cl1	0.68929 (13)	0.04557 (4)	0.92197 (7)	0.0738 (3)	
N1	0.5353 (5)	0.73926 (13)	0.88443 (19)	0.0595 (7)	
N2	0.8868 (3)	0.42344 (11)	0.86323 (19)	0.0495 (6)	
N3	0.9879 (3)	0.35976 (11)	0.86989 (17)	0.0478 (6)	
O1	0.3541 (4)	0.73510 (11)	0.89719 (19)	0.0807 (7)	
O2	0.6246 (4)	0.79469 (11)	0.87488 (19)	0.0824 (7)	
O3	1.1727 (3)	0.48808 (10)	0.86669 (16)	0.0623 (6)	
O4	1.2997 (3)	0.26460 (10)	0.87161 (18)	0.0659 (6)	
H4	1.2520	0.3044	0.8735	0.099*	
O5	0.4676 (3)	0.40021 (11)	0.80587 (18)	0.0674 (6)	
H5	0.3727	0.4232	0.8281	0.101*	
C1	0.6514 (4)	0.67373 (13)	0.8806 (2)	0.0470 (7)	
C2	0.5546 (4)	0.61164 (14)	0.9009 (2)	0.0548 (8)	
H2	0.4187	0.6113	0.9177	0.066*	
C3	0.6617 (4)	0.54978 (14)	0.8959 (2)	0.0518 (8)	
H3	0.5977	0.5074	0.9108	0.062*	
C4	0.8634 (4)	0.54947 (13)	0.8691 (2)	0.0426 (6)	
C5	0.9558 (4)	0.61350 (14)	0.8499 (2)	0.0521 (7)	
H5A	1.0915	0.6143	0.8327	0.063*	
C6	0.8517 (5)	0.67595 (14)	0.8558 (2)	0.0549 (8)	
H6	0.9156	0.7187	0.8432	0.066*	
C7	0.9882 (4)	0.48478 (14)	0.8649 (2)	0.0443 (6)	
C8	0.8723 (4)	0.30647 (14)	0.8789 (2)	0.0488 (7)	
H8	0.7338	0.3140	0.8806	0.059*	
C9	0.9467 (4)	0.23460 (13)	0.8868 (2)	0.0443 (7)	
C10	1.1506 (4)	0.21677 (14)	0.8820 (2)	0.0499 (7)	
C11	1.2063 (5)	0.14648 (15)	0.8879 (3)	0.0635 (9)	
H11	1.3419	0.1344	0.8842	0.076*	
C12	1.0660 (5)	0.09492 (15)	0.8992 (2)	0.0607 (8)	
H12	1.1058	0.0480	0.9025	0.073*	
C13	0.8651 (4)	0.11224 (14)	0.9058 (2)	0.0503 (7)	
C14	0.8072 (4)	0.18097 (14)	0.8996 (2)	0.0497 (7)	
H14	0.6711	0.1922	0.9042	0.060*	
C15	0.4413 (6)	0.38939 (17)	0.6953 (3)	0.0850 (11)	
H15A	0.3767	0.4298	0.6636	0.127*	
H15B	0.3587	0.3485	0.6830	0.127*	
H15C	0.5704	0.3824	0.6637	0.127*	
H2A	0.7515 (16)	0.4223 (17)	0.856 (2)	0.080*	

### Atomic displacement parameters (Å^2^)

**Table:**

	*U*^11^	*U*^22^	*U*^33^	*U*^12^	*U*^13^	*U*^23^
Cl1	0.0638 (6)	0.0528 (5)	0.1047 (7)	−0.0066 (4)	−0.0002 (5)	0.0189 (4)
N1	0.071 (2)	0.0480 (16)	0.0598 (16)	0.0150 (14)	0.0003 (14)	−0.0022 (12)
N2	0.0387 (13)	0.0391 (12)	0.0704 (16)	0.0081 (12)	−0.0020 (12)	0.0003 (11)
N3	0.0466 (15)	0.0393 (13)	0.0575 (15)	0.0082 (11)	−0.0014 (11)	0.0002 (11)
O1	0.0651 (17)	0.0686 (15)	0.1087 (19)	0.0264 (13)	0.0099 (14)	−0.0013 (12)
O2	0.099 (2)	0.0440 (13)	0.1044 (19)	0.0093 (13)	0.0000 (15)	0.0054 (12)
O3	0.0380 (13)	0.0549 (12)	0.0939 (16)	0.0046 (10)	0.0008 (11)	−0.0051 (11)
O4	0.0451 (13)	0.0489 (12)	0.1037 (17)	0.0004 (10)	0.0008 (12)	0.0042 (12)
O5	0.0445 (13)	0.0623 (14)	0.0952 (17)	0.0103 (10)	−0.0024 (11)	−0.0156 (11)
C1	0.053 (2)	0.0405 (15)	0.0476 (16)	0.0108 (13)	−0.0011 (14)	−0.0005 (12)
C2	0.0428 (17)	0.0491 (17)	0.073 (2)	0.0058 (14)	0.0056 (15)	−0.0036 (15)
C3	0.0441 (18)	0.0399 (15)	0.071 (2)	0.0018 (13)	0.0055 (15)	−0.0016 (13)
C4	0.0378 (16)	0.0421 (15)	0.0479 (16)	0.0023 (12)	−0.0013 (12)	−0.0007 (12)
C5	0.0388 (17)	0.0523 (17)	0.0653 (19)	−0.0020 (14)	0.0007 (14)	0.0024 (14)
C6	0.055 (2)	0.0425 (16)	0.067 (2)	−0.0003 (14)	−0.0032 (16)	0.0048 (14)
C7	0.0377 (17)	0.0446 (15)	0.0505 (17)	0.0030 (13)	−0.0018 (13)	−0.0017 (13)
C8	0.0412 (17)	0.0454 (16)	0.0599 (18)	0.0082 (13)	0.0017 (14)	0.0011 (13)
C9	0.0389 (16)	0.0425 (15)	0.0514 (17)	0.0040 (12)	−0.0009 (13)	0.0010 (12)
C10	0.0435 (18)	0.0461 (16)	0.0600 (18)	0.0012 (14)	−0.0002 (14)	0.0021 (14)
C11	0.0441 (18)	0.0485 (17)	0.098 (2)	0.0127 (15)	0.0008 (16)	0.0038 (17)
C12	0.058 (2)	0.0437 (17)	0.080 (2)	0.0052 (15)	−0.0042 (16)	0.0064 (15)
C13	0.0495 (18)	0.0420 (15)	0.0594 (18)	−0.0005 (13)	−0.0024 (14)	0.0065 (13)
C14	0.0379 (17)	0.0496 (16)	0.0616 (18)	0.0062 (13)	0.0014 (13)	0.0011 (14)
C15	0.092 (3)	0.076 (2)	0.087 (3)	0.000 (2)	0.006 (2)	−0.003 (2)

### Geometric parameters (Å, °)

**Table:**

Cl1—C13	1.735 (3)	C4—C5	1.384 (3)
N1—O2	1.215 (3)	C4—C7	1.482 (4)
N1—O1	1.217 (3)	C5—C6	1.374 (4)
N1—C1	1.464 (3)	C5—H5A	0.93
N2—C7	1.344 (3)	C6—H6	0.93
N2—N3	1.384 (3)	C8—C9	1.453 (3)
N2—H2A	0.899 (10)	C8—H8	0.93
N3—C8	1.275 (3)	C9—C14	1.387 (4)
O3—C7	1.225 (3)	C9—C10	1.396 (4)
O4—C10	1.350 (3)	C10—C11	1.386 (4)
O4—H4	0.82	C11—C12	1.360 (4)
O5—C15	1.407 (4)	C11—H11	0.93
O5—H5	0.82	C12—C13	1.375 (4)
C1—C2	1.368 (4)	C12—H12	0.93
C1—C6	1.371 (4)	C13—C14	1.362 (3)
C2—C3	1.374 (3)	C14—H14	0.93
C2—H2	0.93	C15—H15A	0.96
C3—C4	1.386 (4)	C15—H15B	0.96
C3—H3	0.93	C15—H15C	0.96
			
O2—N1—O1	123.6 (3)	N2—C7—C4	116.0 (2)
O2—N1—C1	118.3 (3)	N3—C8—C9	123.2 (3)
O1—N1—C1	118.0 (3)	N3—C8—H8	118.4
C7—N2—N3	121.0 (2)	C9—C8—H8	118.4
C7—N2—H2A	121 (2)	C14—C9—C10	118.4 (2)
N3—N2—H2A	118 (2)	C14—C9—C8	118.1 (2)
C8—N3—N2	114.0 (2)	C10—C9—C8	123.5 (3)
C10—O4—H4	109.5	O4—C10—C11	117.2 (3)
C15—O5—H5	109.5	O4—C10—C9	123.6 (2)
C2—C1—C6	121.9 (3)	C11—C10—C9	119.2 (3)
C2—C1—N1	118.5 (3)	C12—C11—C10	121.1 (3)
C6—C1—N1	119.6 (3)	C12—C11—H11	119.4
C1—C2—C3	118.9 (3)	C10—C11—H11	119.4
C1—C2—H2	120.6	C11—C12—C13	120.0 (3)
C3—C2—H2	120.6	C11—C12—H12	120.0
C2—C3—C4	121.1 (3)	C13—C12—H12	120.0
C2—C3—H3	119.4	C14—C13—C12	119.8 (3)
C4—C3—H3	119.4	C14—C13—Cl1	121.1 (2)
C5—C4—C3	118.1 (2)	C12—C13—Cl1	119.1 (2)
C5—C4—C7	118.2 (2)	C13—C14—C9	121.5 (3)
C3—C4—C7	123.7 (2)	C13—C14—H14	119.3
C6—C5—C4	121.6 (3)	C9—C14—H14	119.3
C6—C5—H5A	119.2	O5—C15—H15A	109.5
C4—C5—H5A	119.2	O5—C15—H15B	109.5
C1—C6—C5	118.4 (3)	H15A—C15—H15B	109.5
C1—C6—H6	120.8	O5—C15—H15C	109.5
C5—C6—H6	120.8	H15A—C15—H15C	109.5
O3—C7—N2	122.9 (2)	H15B—C15—H15C	109.5
O3—C7—C4	121.0 (2)		

### Hydrogen-bond geometry (Å, °)

**Table:**

*D*—H···*A*	*D*—H	H···*A*	*D*···*A*	*D*—H···*A*
O4—H4···N3	0.82	2.04	2.745 (3)	144
O4—H4···O5^i^	0.82	2.47	2.930 (3)	116
O5—H5···O3^ii^	0.82	1.88	2.692 (3)	171
N2—H2A···O5	0.899 (10)	2.016 (13)	2.888 (3)	163 (3)

Symmetry codes: (i) *x*+1, *y*, *z*; (ii) *x*−1, *y*, *z*.
